# Concurrent gemcitabine and 3D radiotherapy in patients with stage III unresectable non-small cell lung cancer

**DOI:** 10.1186/1748-717X-9-190

**Published:** 2014-08-29

**Authors:** Gerald SMA Kerner, Leon FA van Dullemen, Erwin M Wiegman, Joachim Widder, Edwin Blokzijl, Ellen M Driever, John WG van Putten, Jeroen JW Liesker, Tineke EJ Renkema, Remge M Pieterman, Marc JF Mertens, Thijo JN Hiltermann, Harry JM Groen

**Affiliations:** University of Groningen and Department of Pulmonary Diseases, University Medical Center Groningen, Hanzeplein 1, P.O. Box 30.001, Groningen, 9700 RB The Netherlands; University of Groningen and Department of Radiation Oncology, University Medical Center Groningen, Groningen, The Netherlands; Martini Hospital, Groningen, The Netherlands; Scheper Hospital, Emmen, The Netherlands; Refaja Hospital, Stadskanaal, The Netherlands; Ommelander Hospital Group, Delfzijl, The Netherlands; Wilhelmina Hospital, Assen, The Netherlands

**Keywords:** Gemcitabine, Stage III NSCLC, Radiotherapy, Concurrent chemoradiotherapy

## Abstract

**Background:**

Stage III unresectable non-small cell lung cancer (NSCLC) is preferably treated with concurrent schedules of chemoradiotherapy, but none is clearly superior Gemcitabine is a radiosensitizing cytotoxic drug that has been studied in phase 1 and 2 studies in this setting. The aim of this study was to describe outcome and toxicity of low-dose weekly gemcitabine combined with concurrent 3-dimensional conformal radiotherapy (3D-CRT).

**Patients & methods:**

Treatment consisted of two cycles of a cisplatin and gemcitabine followed by weekly gemcitabine 300 mg/m^2^ during 5 weeks of 3D-CRT, 60 Gy in 5 weeks (hypofractionated-accelerated). Overall survival (OS), progression-free survival (PFS), and treatment related toxicity according to Common Toxicity Criteria of Adverse Events (CTCAE) version 3.0 were assessed.

**Results:**

Between February 2002 and August 2008, 318 patients were treated. Median age was 64 years (range 36–86); 72% were male, WHO PS 0/1/2 was 44/53/3%. Median PFS was 15.5 months (95% confidence interval [CI], 12.9-18.1) and median OS was 24.6 months (95% CI., 21.0-28.1). Main toxicity (CTCAE grade ≥3) was dysphagia (12.6%), esophagitis (9.6%), followed by radiation pneumonitis (3.0%). There were five treatment related deaths (1.6%), two due to esophagitis and three due to radiation pneumonitis.

**Conclusion:**

Concurrent low-dose gemcitabine and 3D-CRT provides a comparable survival and toxicity profile to other available treatment schemes for unresectable stage III.

## Introduction

At presentation approximately 30% of patients with non-small-cell lung cancer (NSCLC) have locally advanced (stage III) disease [1]. Median overall survival for clinically staged NSCLC stage IIIA is 14 months and for stage IIIB 10 months with five-year survival of 19% and 7%, respectively [2]. For good performance patients with unresectable stage III disease, the treatment of choice is concurrent chemoradiotherapy [3, 4]. Different treatment modalities are used in practice, but none is clearly superior to others [5–11].

Gemcitabine is among the strongest radiosensitizing drugs available, but its use in lung cancer has been limited due to substantial toxicity when combining full-dose gemcitabine with radiotherapy in treatment of stage III NSCLC [12, 13]. Excess rates of radiation pneumonitis have been reported using gemcitabine in chemoradiation for lung cancer in earlier studies that had used 2D radiotherapy [14, 15]. Previously, a phase I trial was conducted at our institution establishing a safe schedule of concurrent weekly 300 mg/m^2^ gemcitabine and 3D-CRT [16]. These results were confirmed in a phase II trial at our institution [17].

Here we report survival time and treatment related toxicity of this treatment regimen in a consecutive cohort of patients receiving gemcitabine-based concurrent chemoradiotherapy for stage III unresectable NSCLC over a period of 7 years. A subgroup analysis of patients aged >70 years was planned a priori.

## Materials and methods

### Patient selection

From February 2002 until December 2009, consecutive patients with stage III unresectable NSCLC who were treated with concurrent chemoradiotherapy with gemcitabine as radiosensitizer referred to one radiotherapy department from 6 hospitals in the northern part of the Netherlands were studied. Staging was performed by whole body ^18^ F-FDG-PET, contrast enhanced chest CT, bronchoscopy, endoscopic ultrasound with fine-needle biopsy and mediastinoscopy. Treatment-related decisions were made during the weekly multidisciplinary meetings. The decision for treating these patients with this scheme, which was regarded as the standard treatment protocol for NSCLC stage III, was based on physical condition (performance status according to WHO), co-morbidity, and expected radiotherapy dose–volume constraints.

### Induction chemotherapy

Chemotherapy consisted of two cycles of cisplatin 75 mg/m^2^ on day 1 and gemcitabine 1125 mg/m^2^ administered intravenously on day 1 and 8 of each 21-day cycle. This dose of gemcitabine provides the same dose intensity as the older 3 out of 4 week schedule. Anti-emetics were ondansetron 8 mg twice daily on days 1 and 2, and dexamethasone 8 mg twice a day on days 1 and 2 of each cycle and aprepitant 125 mg on day 1 and 80 mg on days 2 and 3. The interval between the first dose of induction chemotherapy and the start of radiotherapy was 6 weeks.

### Radiotherapy with gemcitabine as sensitizer

Patients received 3D-CRT to a total dose of 60 Gy, administered over 5 weeks in once daily fractions of 2.4 Gy five days per week, together with once weekly gemcitabine 300 mg/m^2^. Gemcitabine was omitted when leucocytes were below 3.10^9^/L or platelets below 100.10^9^/L. The gross target volume (GTV) was delineated on the planning-CT and included the primary tumor and enlarged FDG-avid, or pathologically proven lymph nodes. Until 2005, tumor motion was determined with fluoroscopy and respective margins were added to the GTV, thereafter, an internal target volume (ITV) was individually delineated using a 4D-planning CT. A 5 mm margin was added to arrive at the clinical target volume, another 6 mm were added for the planning target volume (PTV). Radiotherapy was delivered using 6 MV photons, the dose was specified at the isocenter and was corrected for pulmonary heterogeneity. The total radiation dose administered corresponded to an equivalent dose of 62 Gy if it were given in 2 Gy daily fractions.

### Dose constraints

The spinal cord dose was constrained to 50 Gy. The mean lung dose (MLD) should not exceed 20 Gy (uncorrected for the slightly higher dose per fraction; this would equal 21.6 Gy converted to 2Gy/fraction equivalent dose using an alpha/beta of 3Gy in the linear quadratic formula). The V_20_ (the volume of the total lung volume receiving ≥ 20 Gy) was constrained to 30% (this value is challenging to recalculate using the linear-quadratic formula, but will correspond to a value about 8% higher if given in 2Gy daily fractions).

For patients with high-volume disease, a proof-planning was made and evaluated for feasibility by the radiation oncologist. If necessary, the radiation dose was adapted. If the pulmonary dose constraints were still considerably exceeded after radiation dose reduction, patients were excluded from this protocol.

### Treatment evaluation

Complete blood cell counts were performed on days 1, 8, and 22 of each induction chemotherapy cycle. On day 1 of each cycle, patient evaluation also included liver and renal functions, performance status, and toxicity scoring. During radiotherapy, complete blood cell counts and toxicity evaluation were performed. Two months after completion of treatment a response CT scan was obtained and patients were followed every 3 months with physical examination, laboratory tests and chest x-ray. Disease progression was defined according to RECIST 1.0 criteria [18]. Toxicity (esophagitis, and radiation pneumonitis) related to treatment was retrospectively scored using the Common Toxicity Criteria (CTCAE 3.0) of the National Cancer Institute.

### Statistical analysis

Descriptive statistics were used to characterize clinical features and toxicity. Using the Kaplan-Meier method, overall survival (OS) and progression-free survival (PFS) were calculated from the date of diagnosis until death, loss to follow-up, or first documentation of progressive disease, respectively. A subgroup analysis in patients aged over 70 years was performed to evaluate the outcome in elderly patients. Cox regression analysis was performed with the variables age, smoking, gender, histology, radiation dose, WHO performance score and PTV. A p-value of < 0.05 was considered statistically significant. All calculations were performed using SPSS 20.0 (International Business Machines Corp, Armonk, NY, USA).

## Results

Three hundred and eighteen subsequent patients receiving concurrent chemoradiotherapy with gemcitabine as a radiosensitizer were studied. Median age was 64 years (range 36–86); male/female was 72/28%; WHO PS 0/1/2 was 44, 53 and 3%. A total of 93 patients were aged ≥ 70.

### Chemotherapy

Two hundred and forty-two patients (76%) received cisplatin and gemcitabine as induction, 42 (13%) received carboplatin and gemcitabine, and 9 patients (3%) received other schedules. Twenty five (8%) patients did not receive induction chemotherapy. A total of 259 patients (81%) received all 5 weekly doses of gemcitabine during radiotherapy, 244 (76%) received both full dose gemcitabine and full dose radiotherapy. Fifty-one patients (16%) received between 1 and 4 gemcitabine administrations weekly, mainly due to neutropenia and thrombocytopenia. For the remaining 8, no information was available. Patient characteristics are detailed in Table 1. Interestingly, advanced age did not lead to decreasing the concomitant gemcitabine dose (Table 2).

**Table 1:** **Patient characteristics**

	Number of patients	
	(***N*** = 318)	%
Age	64 (36–86)	
Male/Female ratio	229/89	72/28
WHO 0	129	44
1	151	53
2	10	3
Unknown	28	
Histology		
Adenocarcinoma	68	21.4
Squamous cell	157	49.4
NSCLC-NOS	93	29.2
Smokers		
None	9	3
Current	163	51
Former	109	34
Unknown	37	12
Induction treatment		
Cisplatin/gemcitabine	242	76
Carboplatin/gemcitabine	42	13
Platinum/pemetrexed	4	1
Other	5	2
No induction	25	8
Radiotherapy dose received		
30-59 Gy	34	11
60 Gy	284	89
>60 Gy	0	0
During chemoradiotherapy:		
Received 5 gemcitabine cycles		
and 60 Gy	244	76
Received 1–4 gemcitabine cycles		
and/or received less		
then 60 Gy	74	24

**Table 2:** **Number of cycles of weekly gemcitabine with respect to age in stage III NSCLC**

	Age < 70		Age ≥ 70		Total	
1	1	(<1%)	0		1	(<1%)
2	7	(3%)	3	(3%)	10	(3%)
3	9	(4%)	7	(8%)	16	(5%)
4	13	(6%)	11	(12%)	24	(8%)
5	188	(84%)	71	(76%)	259	(84%)

From 8 patients the weekly gemcitabine dosage was not specified.

Seven patients (2%) had a tumor resection after chemoradiotherapy.

### Radiotherapy

The radiotherapy dose was 60 Gy in 284 patients (89%) while less than 60 Gy (range, 29 to 58 Gy) was administered in 34 patients (11%). These 34 patients included 17 patients with increased risk of radiation pneumonitis due to increased V_20_ and 8 patients who stopped treatment due to radiation related toxicity including 3 patients with CTC grade 3 esophagitis. Nine patients received less than 60 Gy for undocumented reasons.

The median PTV was 431 cc with the 90^th^ percentile at 734 cc. Median V_20_ was 22.7% and the 90^th^ percentile was 31.0%. In patients aged ≥ 70, median PTV was not different at 411 cc with the 90^th^ percentile at 702 cc. Median V_20_ was 23%, with the 90^th^ percentile at 30.6%.

### Survival

**Figure 1:**
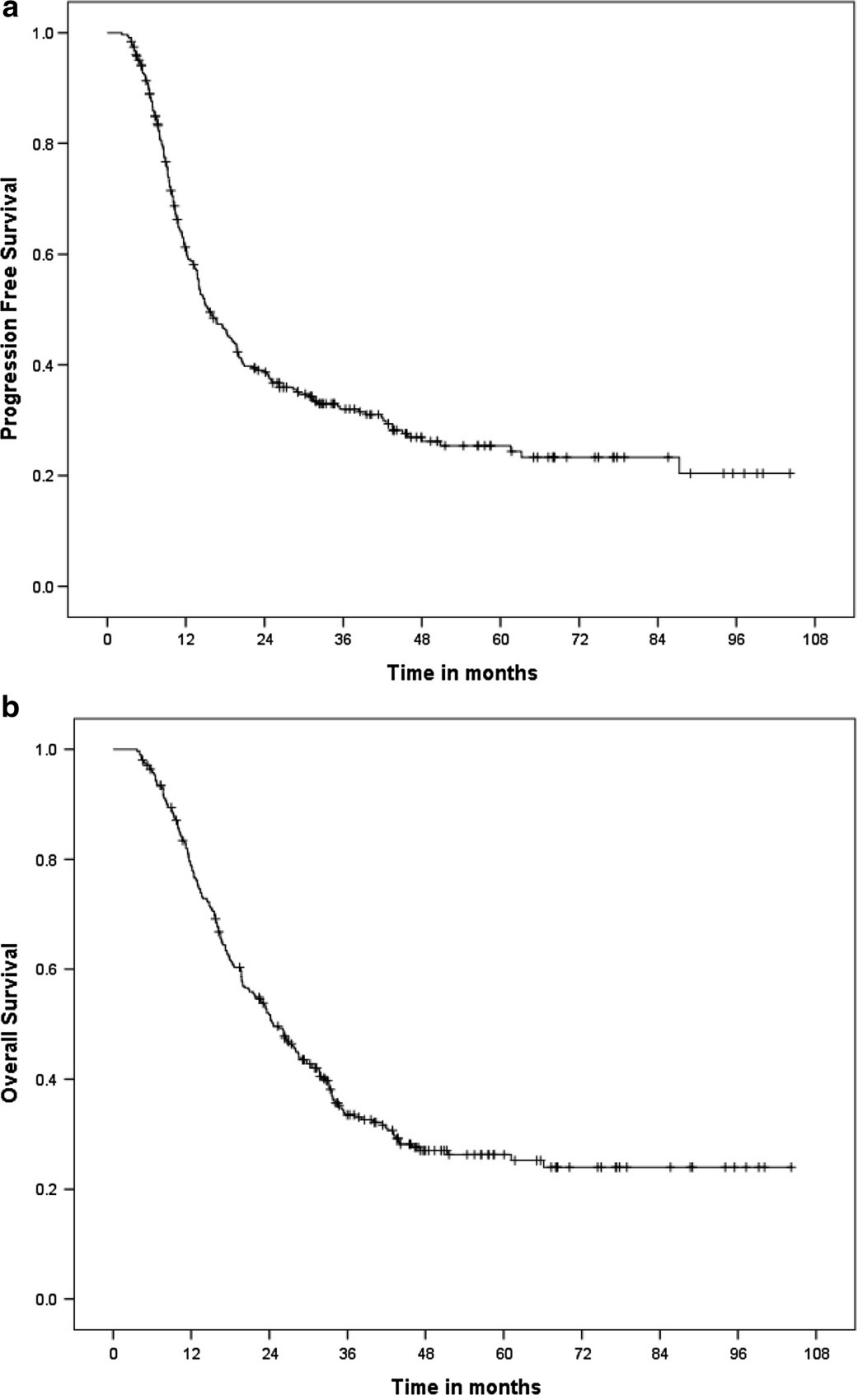
**Survival of 318 patients treated with gemcitabine and 3D concurrent radiotherapy. a**. Median Progression Free Survival was 15.5 months (95% CI., 12.9-18.1). **b**. Median Overall Survival was 24.6 months (95% CI., 21.0-28.1) with a 5-year survival of 26%.

Median PFS was 15.5 months (95% CI., 12.9-18.1) (Figure 1a) and median OS was 24.6 months (95% CI., 21.0-28.1) with a 5-year survival of 26% (Figure 1b). In the 244 patients who completed full concurrent treatment, median survival was 26.3 months (95% CI., 21.9-30.6 months) with a 5-year survival of 27%. In patients aged ≥ 70, median PFS was 18.7 (95% CI., 10.0-27.4) and OS was 26.2 months (95% CI., 19.0-33.4) with a 19% 5 year survival rate.

Using univariate Cox analysis, current smoking at diagnosis, squamous cell histology, higher WHO performance score, male gender and larger PTV, were all identified as negative factors influencing survival. In multivariate Cox analysis, current smoking, male gender and larger PTV remained as negative factors influencing survival.

### Toxicity

Most common toxicities were CTC grade 2 esophagitis (13.6%), and CTC grade 2 radiation pneumonitis (17.7%) (Table 3). CTC grade ≥3 esophagitis was seen in 9.7% with 2 CTC grade 5 events. One patient had an esophageal ulcerative stenosis which resulted in death due to a massive hemorrhage. The second patient received a stent for an esophageal stenosis. Later this patient developed an esophageal-bronchial fistula and died 22 months later. Three percent (*N* = 10) of the patients had CTC grade ≥3 radiation pneumonitis. Three patients had a grade 5 event (all aged above 70 years). For these three patients, the PTV was 508, 600 and 578 cc (the 75% percentile of the whole cohort was 576 cc), with a V_20_ of 16, 34, and 30%, respectively. The first patient had no induction chemotherapy, the two others were treated with induction carboplatin and gemcitabine. In patients who completed the full chemoradiotherapy (*N* = 244), 7% had CTC grade ≥3 esophagitis (including 1 CTC grade 5 event), and 4% CTC grade ≥3 radiation pneumonitis (including 2 CTC grade 5 events). In patients aged 70 and older (*N* = 93), 9 patients (9.7%) had CTC grade 3 esophagitis, 4 patients (4.3%) had CTC grade 3 radiation pneumonitis, and three patients (3.2%) died due to pneumonitis. The other patients died due to progressive disease.

**Table 3:** **Radiation toxicity CTC ≥ 2 in patients with stage III NSCLC treated with concurrent gemcitabine and 3D radiotherapy**

Toxicity	Total		Age < 70		Age ≥ 70	
	N = 318	%	N = 225	%	N = 93	%
Esophagitis						
CTC 2	43	13.6	32	14.3	11	11.8
CTC 3	29	9.1	20	8.9	9	9.7
CTC 4	1	0.3	1	0.4	0	
CTC 5	2	0.6	2	0.9	0	
Radiation pneumonitis						
CTC 2	52	17.7	38	17.0	14	15.1
CTC 3	6	2.0	2	0.9	4	4.3
CTC 4	1	0.3	1	0.4	0	
CTC 5	3	0.9	0		3	3.2

## Discussion

In this paper, we described outcome and toxicity of weekly gemcitabine and 5 weeks of 3D-CRT for stage III NSCLC. The overall median survival was 24,6 months. Although comparisons between uncontrolled single institution series should be interpreted with great caution due to possible variation in patient selection criteria, in other concurrent chemoradiotherapy regimens median survival was between 15.3 and 26 months [5–10, 19–21]. In addition, esophageal toxicity and radiation pneumonitis was also in the same range as other recent studies with concurrent chemoradiation [5–10, 19–21].

In 2003 it had been shown that concurrent low-dose gemcitabine and 3D-CRT had acceptable toxicity [16]. Subsequently, a phase 2 study showed a median PFS of 12.4 months and OS of 21.6 months with acceptable toxicity [17]. This was the reason to adopt the here reported regimen as our protocol in the region, because we were unable to recognize excessive toxicity as reported by others, in our patients. For instance, in a study from Blanco et al., drug dosage was adapted due to toxicity while other studies have been closed due to unacceptable toxicity profiles (especially pulmonary toxicity) [15, 22, 23]. Price et al. investigated gemcitabine at a lower dosage (100 mg/m^2^) combined with a lower radiation dosage (55 Gy) given in a shorter time span (4 weeks) in a study that was prematurely closed due to slow accrual. There were 2 deaths in the gemcitabine arm due to acceleration of pre-existing interstitial lung disease [24]. Of note, in that study a daily fraction-dose of 2.75 Gy was given and the lung-dose-constraints had not been adapted for this higher dose per fraction. To our knowledge, there are no further reports using hypofractionated accelerated radiotherapy combined with gemcitabine.

The main factors that are important concerning pulmonary toxicity (i.e. radiation pneumonitis) during chemoradiation with concurrent low-dose gemcitabine are not completely understood. Lung dose, the use of conformal radiotherapy and the timing of the gemcitabine dosage are primary candidates. The CALGB 30105 trial showed that a V_20_ of 40% was associated with significant grade 3–5 pulmonary toxicity [25], which would be expected with any other combination treatment, even with radiotherapy alone. We observed no unexpected pulmonary toxicity rates, because at our institute, the constraint for V_20_ was set at a rather conservative 30% (uncorrected for the slightly higher daily fraction dose of 2.4 Gy) and indeed the vast majority (90%) of treated patients had a V_20_ lower than 30%. Elective nodal irradiation was completely foregone in the first year of this study, with no ensuing statistically significant toxicity differences. However, all three patients in our study who died due to radiation pneumonitis were aged above 70 – and had V_20_ of 16, 34 and 30%, respectively. The CALGB study also identified older age to be associated with increased pulmonary toxicity [25].

Also for esophageal toxicity, radiation technique had been shown to be critical. In a phase 1 study, which initially started using 2D conformal techniques, but halfway switched to 3D techniques, the percentage of the esophagus irradiated to 60 Gy dropped from 68 to 18%, and grade 2 esophagitis dropped from 5/10 patients to 2/14 patients [14].

Administration of gemcitabine more frequently than once weekly was also associated with increased toxicity. One study administered gemcitabine twice weekly (50 mg/m^2^) with 3D radiotherapy with elective nodal irradiation of the mediastinum [22]. Of note, the PTV’s in that study were three times as large as ours due to elective nodal irradiation. Due to unacceptable toxicity, the gemcitabine dose was reduced to 35 mg/m^2^, but even after this reduction, still more severe CTC grade ≥ 3 esophageal and pulmonary toxicity was observed compared to our study. Our once weekly schedule featured 3 (for 50 mg/ m^2^) to 4.3 times (for 35 mg/ m^2^) the cumulative weekly gemcitabine dose [22]. This suggests that timing of drug administration may trigger toxicity, as was also demonstrated in our previous phase 1 and 2 clinical trials.

There were a total of 5 (1.6%) non-hematological grade 5 events in our study, which is comparable to other mainstream treatment regimens [6, 7, 10, 20].

## Conclusions

Treatment of patients with unresectable stage III NSCLC with cisplatin and gemcitabine followed by concurrent gemcitabine and 3D conformal radiotherapy was safe and yielded effectiveness and toxicity rates comparable with other drugs in our hands. These results are very likely due to conservative radiation dose-constraints and 3D-conformal radiotherapy avoiding irradiation of excessive volumes of uninvolved mediastinal areas. Although age was not a factor influencing survival and the absolute incidence at 3% grade 5 pneumonitis in patients older than 70 years was what could be expected, patients above 70 years of age should be selected with great caution for this regimen.
